# Pre-hospital care & interfacility transport of 385 COVID-19 emergency patients: an air ambulance perspective

**DOI:** 10.1186/s13049-020-00789-8

**Published:** 2020-09-22

**Authors:** Peter Hilbert-Carius, Jörg Braun, Fikri Abu-Zidan, Jörn Adler, Jürgen Knapp, Didier Dandrifosse, Désirée Braun, Urs Pietsch, Patrick Adamczuk, Leif Rognås, Roland Albrecht

**Affiliations:** 1 Department of Anaesthesiology, Intensive Care and Emergency Medicine, Pain Therapy, BG-Klinikum Bergmannstrost Halle (Saale), Merseburgerstr 165, 06112 Halle (Saale), Germany; 2 DRF-Luftrettung (German Air Rescue), HEMS Christoph 84 and Christoph 85 Halle (Saale), Halle (Saale), Germany; 3 Faculty of Medicine -Wissenschaftlicher Arbeitskreis der DRF Stiftung Luftrettung (German Air Rescue) gemeinnützige AG, Filderstadt, Germany; 4 Department of Surgery, College of Medicine and Health Science, UAE University, Al-Ain, United Arab Emirates; 5 LAR-Luxembourg Air Rescue, Sandweiler, Luxembourg; 6 Department of Anaesthesiology and Pain Medicine, Inselspital, Bern University Hospital, University of Bern, Bern, Switzerland; 7 Swiss Air Rescue Rega, Zürich, Switzerland; 8 Department of Anaesthesiology and Intensive Care Medicine, Cantonal Hospital St. Gallen, St. Gallen, Switzerland; 9 Department of Anaesthesia, Aarhus University Hospital, Aarhus, Denmark; 10 Den Landsdækkende Akutlægehelikopterording (Danish Air Ambulance), Aarhus, Denmark

**Keywords:** COVID-19, Infection, Medical staff, Helicopter, Fixed wing aircraft, Prevention, Emergency medicine

## Abstract

**Background:**

COVID-19, the pandemic caused by the severe acute respiratory syndrome coronavirus-2, is challenging healthcare systems worldwide. Little is known about problems faced by emergency medical services—particularly helicopter services—caring for suspected or confirmed COVID-19 patients. We aimed to describe the issues faced by air ambulance services in Europe as they transport potential COVID-19 patients.

**Methods:**

Nine different HEMS providers in seven different countries across Europe were invited to share their experiences and to report their data regarding the care, transport, and safety measures in suspected or confirmed COVID-19 missions. Six air ambulance providers in six countries agreed and reported their data regarding development of special procedures and safety instructions in preparation for the COVID-19 pandemic. Four providers agreed to provide mission related data. Three hundred eighty-five COVID-19-related missions were analysed, including 119 primary transport missions and 266 interfacility transport missions.

**Results:**

All providers had developed special procedures and safety instructions in preparation for COVID-19. Ground transport was the preferred mode of transport in primary missions, whereas air transport was preferred for interfacility transport. In some countries the transport of COVID-19 patients by regular air ambulance services was avoided. Patients in interfacility transport missions had a significantly higher median (range) NACA Score 4 (2-5) compared with 3 (1-7), needed significantly more medical interventions, were significantly younger (59.6 ± 16 vs 65 ± 21 years), and were significantly more often male (73% vs 60.5%).

**Conclusions:**

All participating air ambulance providers were prepared for COVID-19. Safe care and transport of suspected or confirmed COVID-19 patients is achievable. Most patients on primary missions were transported by ground. These patients were less sick than interfacility transport patients, for whom air transport was the preferred method.

## Background

COVID-19, the pandemic caused by the severe acute respiratory syndrome (SARS) coronavirus-2 (SARS-CoV-2), is challenging healthcare systems worldwide. In some countries, the number of serious cases has exceeded the resources available and the health care system’s ability to cope. By definition this is a disaster situation, and the SARS-Cov-2 outbreak has been referred to as a mass casualty incident of the highest degree [1, 2].

The COVID-19 virus is highly contagious and can survive for up to 5 days on a variety of surfaces [3, 4]. This is a major problem for healthcare providers who have close contact with COVID-19 patients for long periods. This includes the crews of ground emergency medical services (EMS) and helicopter emergency medical services (HEMS). These services have been extremely busy, with multiple consecutive transport missions.

The healthcare providers and transferred patients are at high risk of infection if the ambulances and aircrafts are insufficiently disinfected before the next mission [5]. Possible ways of infection include respiratory droplets / aerosols, direct / indirect contact with contaminated secretions or surfaces, and medical interventions (e.g. airway management, suction of secretion, CPR) which increase the risk of virus transmission [1, 5–8]. Therefore, a strong hygiene concept, proper personal protective equipment (PPE), standard operating procedures (SOPs), and occasional access to special equipment are of utmost importance when managing patients with suspected or confirmed COVID-19 infection. It is very important to clean and disinfect the ambulances and aircrafts upon arrival at their final home base [9].

Until now, data on COVID-19 patients’ care and transport by air ambulance systems across Europe are very limited [10]. We aimed to describe how six different HEMS/air ambulance systems based in Europe cared for patients with confirmed or suspected COVID-19.

## Methods

### Settings

Nine physician staffed air ambulance providers in seven European countries were invited to participate in the study, to give information, and to share their experiences when transporting COVID-19 patients (see Figure S1 and Table S1 regarding information of the invited and participating countries). In the light of the spreading of the SARS-CoV-2 across Europe, the invited air ambulance providers were asked to provide data regarding the development of special instructions and safety procedures in preparation for missions with suspected or confirmed COVID-19 patients.

Of the nine invited air ambulance providers, six agreed to participate. The participating providers were based in six European countries and agreed to provide the following information (listed in alphabetic order):

1. **Austria:** The ARA-Air Rescue Austria, which uses only helicopters for air ambulance service, provided data regarding preparation, special procedures and safety instruction for COVID-19-related missions.
2. **Denmark:** The Danish Air Ambulance mainly uses helicopters for patient transport, but occasionally transfers patients in road ambulances. It provided data regarding preparation, special procedures and safety instructions for COVID-19-related missions. The database of the service was used to identify COVID-19-related missions.
3. **Germany:** The DRF-Luftrettung (German air rescue) service, which mainly uses helicopters and to a minor degree fixed-wing aircraft, provided data regarding preparation, special procedures and safety instructions for COVID-19-related missions. The database of the service was used to identify COVID-19-related missions.
4. **Luxembourg:** The LAR-Luxembourg Air Rescue service, which uses helicopters and fixed-wing aircraft, provided data regarding preparation, special procedures and safety instructions for COVID-19-related missions. The service’s database was analysed to identify COVID-19-related missions.
5. **Norway:** The Norwegian Air Ambulance service, which uses helicopters and fixed-wing aircraft, provided data regarding preparation, special procedures and safety instructions for COVID-19-related missions.
6. **Switzerland:** The Swiss Air Rescue service Rega, which uses helicopters and fixed-wing aircrafts provided data regarding preparation, special procedures and safety instructions for COVID-19-related missions. The database was used to identify COVID-19-related missions.

### Patient isolation units

Different providers have special patient isolation units (PIUs) in stock for transport of infectious patients, which can also be used to transport COVID-19 patients. The most common PIUs are the EpiShuttel® and the REGA PIU. The Epishuttle® is a CE 1789 compliant reusable single-patient isolation and transport system that can be used in ambulances, helicopters and airplanes. The shuttle can either be used to protect the surroundings from an infectious patient or to protect the patient from the surroundings. During transport, the medical crew does not need to wear full personal protective equipment. The EpiShuttle® is equipped with different ports (operator ports, wire port, ventilator port) and a ventilation system that generates more than 15 air exchanges per hour and can be used with negative or positive pressure inside, depending on who needs to be protected (the patient or the crew). The Rega PIU comprises a flexible safety hull stabilised by arched wires mounted on a hard floor plate. It is maintained under negative pressure by a high-efficiency particulate air filtered ventilation system that uses aircraft power and/or battery power. The PIU barrier performance has proven equal to that provided by protective clothing. Its fixation system allows transportation on any commonly available patient stretcher. The PIU is designed to fit in a fixed-wing ambulance, a medium-sized helicopter, and ground-based ambulances [10].

### Data variables

Four air ambulance providers (see above) agreed to provide data of COVID-19-related missions. Of these COVID-19-related missions, the following variables were studied:

1. **General data:** Age, gender, National Advisory Committee for Aeronautics (NACA) Score, suspected or confirmed SARS-CoV-2 infection.
2. **Mode of transport**: Ground ambulance accompanied by doctor, ground ambulance without HEMS doctor attendance, helicopter transport, fixed-wing aircraft transport, transport to maximum care hospital or basic care hospital, no transport to medical facility.
3. **Medical interventions during missions:** intravenous (i.v.) access, fluid therapy, drug administration, oxygen therapy, non-invasive ventilation (NIV), airway management, lung ultrasound, cardiopulmonary resuscitation (CPR) including the use of an automated chest compression device (ACCD), extracorporeal membrane oxygenation (ECMO).
4. **Hygiene**: face mask for spontaneously breathing patients, use of a filtering face piece (FFP) mask by the crew, use of common surgical face masks by the crew, use of safety glasses/goggles by the crew, use of complete personal protective equipment, final disinfection of the aircraft, use of a PIU.

### Data collection

All missions from February 1, 2020, to April 30, 2020, were included in the retrospective data collection and analysis. The data management systems of the providers agreed to provide mission related information were analysed regarding general data of potential COVID-19-related missions and the above mentioned study variables.

### Statistical analysis

COVID-19-related primary missions were compared with interfacility transport missions regarding general data, mode of transport, medical interventions and hygienic procedures. Data are presented as number (%), mean (SD), or median (range) as appropriate. Fisher’s exact test was used to compare the categorical data of two independent groups, while the Mann-Whitney U test was used to compare continuous or ordinal data of two independent groups. Nonparametric statistical methods were used to compare these groups because these methods compare the ranks and not the crude numbers, without the need for a normal distribution or equal variance of the data, and they protect against small numbers of events. A *p* value of less than 0.05 was considered significant. Statistical analyses were performed using GraphPad Prism 8 for Windows (GraphPad Software 2020, LLC).

### Ethics

The study is in line with the current European general data protection regulation (GDPR).

## Results

During the study period, the participating air ambulances cared for 385 potential COVID-19 patients with a mean age of 61.3 ± 10.8 years and 266 of these were males (69%).The median (range) NACA-Score of these 385 patients was 4 (1-7) and 233 patients (60,5%) had confirmed COVID-19.

### Special procedures and safety instructions

**Fig. 1:**
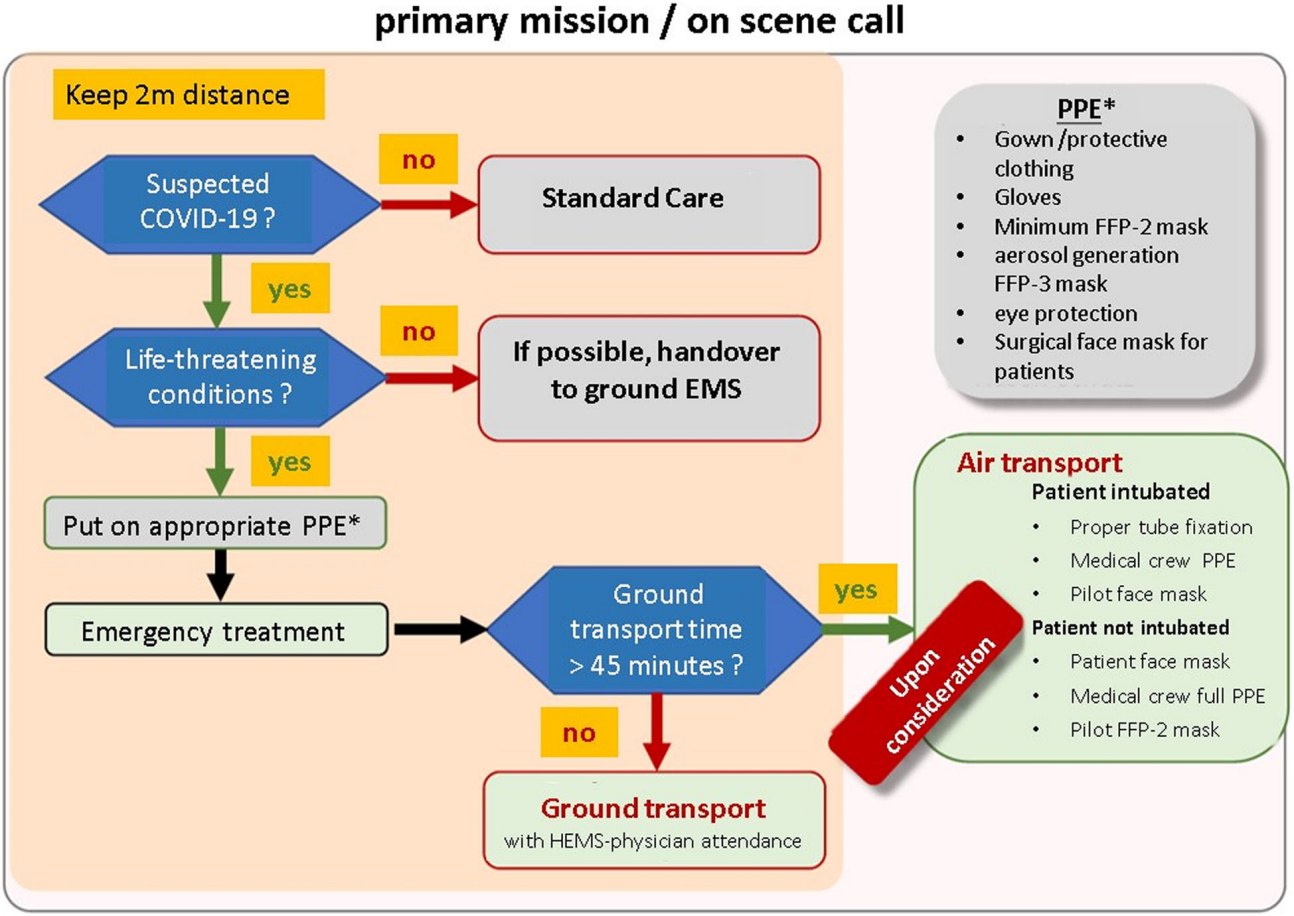
A decision tree for use in evaluating safe transfer during COVID-19-related missions. This one, from the DRF-Luftrettung, is the “Primary mission algorithm for aeromedical crews”

**Fig. 2:**
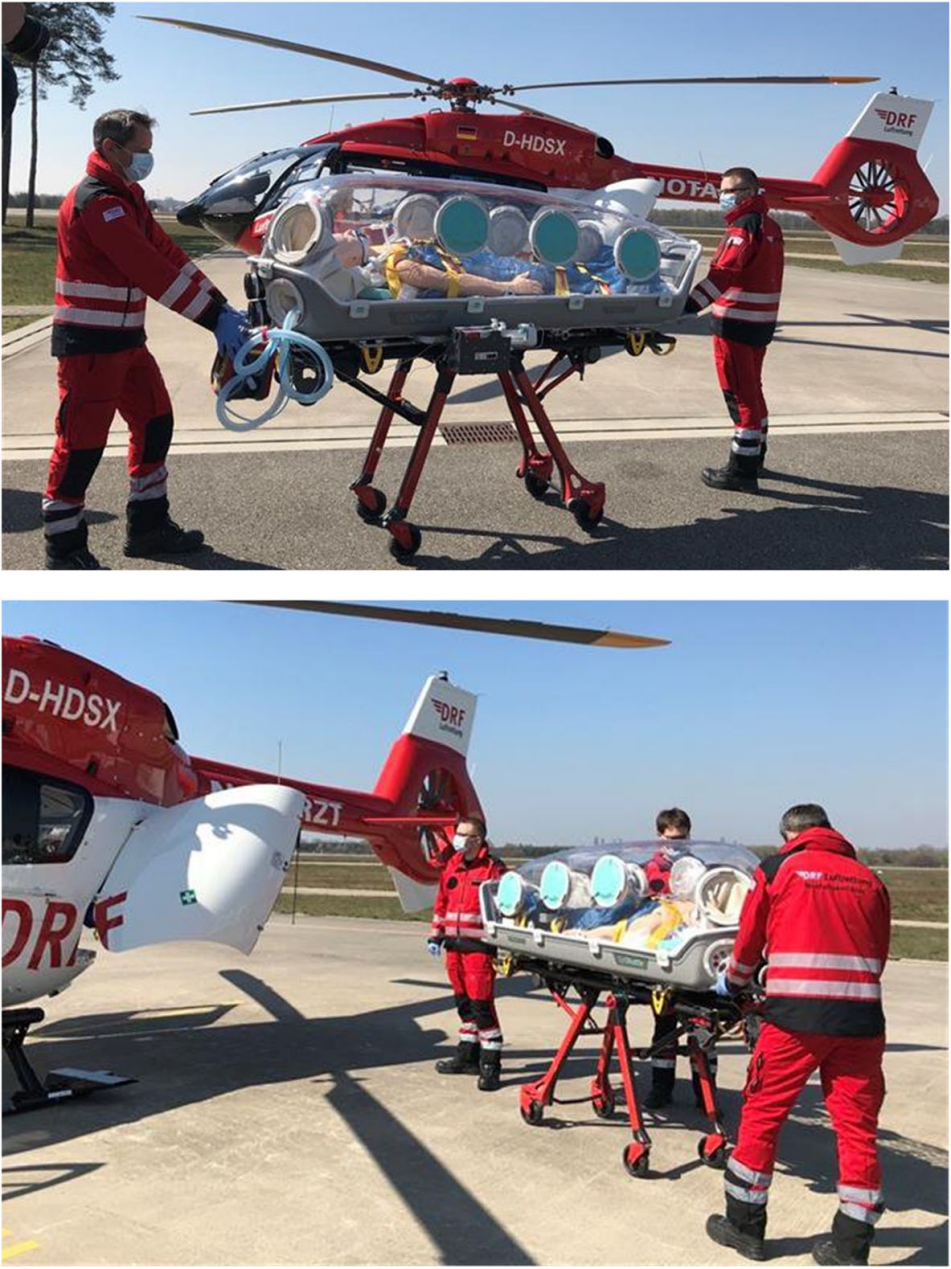
The EpiShuttle® of the DRF-Luftrettung (©DRF-Luftrettung – Germany). The EpiShuttle is used only for interfacility transport. It is fitted into the helicopter (EC 145 / H 145) before the mission. The DRF-Luftrettung has an exemption according to article 71 (1) of regulation (EU) 2018/1139 by the “Luftfahrt-Bundesamt – LBA” (civil aeronautics board of Germany) to use the EpiShuttle in the Eurocopter 145 and Airbus Helicopter 145 rotor wing aircrafts of the company

A majority of the European countries participating had some time to prepare a response to the expected corona pandemic. Accordingly, all participating air ambulance providers used this time to develop special procedures and safety instructions for managing COVID-19 patients (e.g., SOPs regarding intubation, lung ultrasound, CPR, PPE, terminal disinfection). The safety instructions of all providers are very similar (see Fig. 1) and are based on national and international recommendations. Because there was a shortage of proper PPE at the beginning of the pandemic, the safety instructions for PPE use were adjusted according to the availability of protective equipment. During the beginning of the pandemic, PPE has only been used in cases of suspicion or in patients staying in high-risk areas within the last 2 weeks. Sufficient for all missions, PPE were available at the beginning of April. Most missions with any patient contact were carried out with FFP 2/N95 masks and gloves. Depending on further medical interventions and virus transmission risk, a stepwise extension to full PPE (gloves, a fluid-repellent long-sleeved gown or other protective clothing, eye protection, and an FFP 3 mask) was recommended. The aircrafts were supplied with additional full PPE and additional surgical facemasks for spontaneously breathing patients. The aeromedical crews were trained in the proper use of the PPE. For safe transport of patients with confirmed COVID 19 infection, three providers already had special single-patient isolation units (PIUs) and one provider equipped some of the HEMS bases with new PIUs (Fig. 2).

### Transportation of COVID-19 patients

Table 1 shows the COVID-19 mission data over a three-month period (February–April 2020). During that period, the participating air ambulances flew 8340 primary missions and 3029 interfacility transport missions, caring for 385 potential COVID-19 patients. Of these patients, 233 had confirmed COVID-19 infections (60.5%) and 152 had suspected COVID-19 infections (39.5%). For 119 of the patients (31%) it was a primary mission and for 266 it was an interfacility transport (69%). The characteristics of the patients transferred in primary missions and interfacility transport were significantly different (Table 1). Interfacility transport patients had a higher rate of confirmed COVID-19, were younger, were more often male, and were more severely ill (as reflected by the higher NACA score).

**Table 1:** Comparison between primary missions and interfacility transport for COVID-19 related missions by four air ambulance providers from four countries between February 1 and April 30, 2020. Data are presented as mean (SD), median (range), or number (%) as appropriate. *Percentage totals are greater than 100% because 11 patients were transported by both airplane and helicopter

	Primary missions	Interfacility transport	***p*** value
**Total number of missions during study period**	**8340**	**3029**	< 0.001
**General data**			
**Number of patients (% of missions)**	119 (1.4%)	266 (8.8%)	
**Age of patients, mean (SD)**	65.1 (21)	59.6 (6.2)	< 0.01
**Male (%)**	72 (60.5%)	194 (73%)	0.017
**NACA Score, median (range)**	3 (1–7)	4 (2–5)	< 0.001
**Suspected COVID-19**	100 (84%)	52 (19.5%)	< 0.001
**Confirmed COVID-19**	19 (16%)	214 (80.5%)	< 0.001
**Mission-related complications**	2 (1.7%)	4 (1.5%)	0.99
**Mode of transport**			
**Ground ambulance accompanied by doctor**	52 (44%)	42 (16%)*	< 0.001
**Ground ambulance without HEMS doctor attendance**	34 (28.5%)	12 (4.5%)*	< 0.001
**Helicopter transport**	19 (16%)	184 (69%)*	< 0.001
**Fixed-wing aircraft transport**	0	48 (18%)*	< 0.001
**Transport to**			
**maximum care hospital**	64 (54%)	177 (66.5%)	0.022
**basic care hospital**	39 (33%)	10 (4%)	< 0.001
**No transport to medical facility**	14 (11.5%)	1 (0.5%)*	< 0.001
**Medical interventions during mission**			
**i.v. access**	107 (90%)	261 (98%)	< 0.001
**Fluid therapy**	92 (77%)	235 (88%)	< 0.01
**Drug administration**	60 (50%)	225 (84.5%)	< 0.001
**Oxygen therapy**	33 (28%)	205 (77%)	< 0.001
**NIV**	5 (4%)	0	0.002
**Airway management**	18 (15%)	189 (70%)	< 0.001
**Lung ultrasound**	8 (7%)	6 (2%)	0.039
**CPR/use of ACCD**	9 (7.5%)	1 (0.5%)	< 0.001
**ECMO in total**	0	14 (5%)	0.006
**vv-ECMO**	0	9 (3%)	0.062
**va-ECMO**	0	5 (2%)	0.329
**Hygienic procedures**			
**Facemask for spontaneously breathing patients**	95 (80%)	86 (32%)	< 0.001
**FFP 2 (N 95) / FFP 3 (N 99) mask**	82 (68%)	161 (60.5%)	0.137
**Common surgical face mask**	54 (45%)	87 (33%)	0.022
**Safety glasses/goggles**	82 (68%)	251 (94%)	< 0.001
**Complete personal protective equipment**	38 (32%)	176 (66%)	< 0.001
**Final disinfection of aircraft**	118 (99%)	266 (100%)	0.309
**Use of PIU in total**	0	43 (16%)	< 0.001
**EpiShuttle®**	0	23 (8.5%)	< 0.001
**Rega PIU**	0	20 (7.5%)	< 0.001

There were significant differences in the mode of transport used by the air ambulance services for primary and interfacility transport missions. Ground transport was the preferred mode of transport in primary missions (72%), with 28.6% of patients being transported without a physician present. On the other hand, air transport was preferred for interfacility transport (87%). In some countries (Denmark, Norway), transport by regular HEMS was generally avoided. Only COVID-19 patients with a true time-critical condition were transported in an HEMS aircraft, which is a safer and faster method. All other transport missions in both countries were carried out by the Search and Rescue (SAR) service of the armed forces. The 48 long-distance flights (i.e., between European countries and overseas) were carried out by fixed-wing aircraft.

The majority of patients treated needed medical interventions during the missions. Table 1 summarises some of the typical emergency and intensive care interventions. Patients who needed to be transferred to a higher-level medical facility (mainly interfacility transport) were more severely ill and needed significantly more mission-related interventions, except fluid therapy. Non-invasive ventilation (NIV), lung ultrasound and CPR—including ACCD —was used significantly more often in primary missions. During interfacility transport, ECMO was vital to ensure sufficient oxygenation in 14 patients (5.3%).

**Fig. 3:**
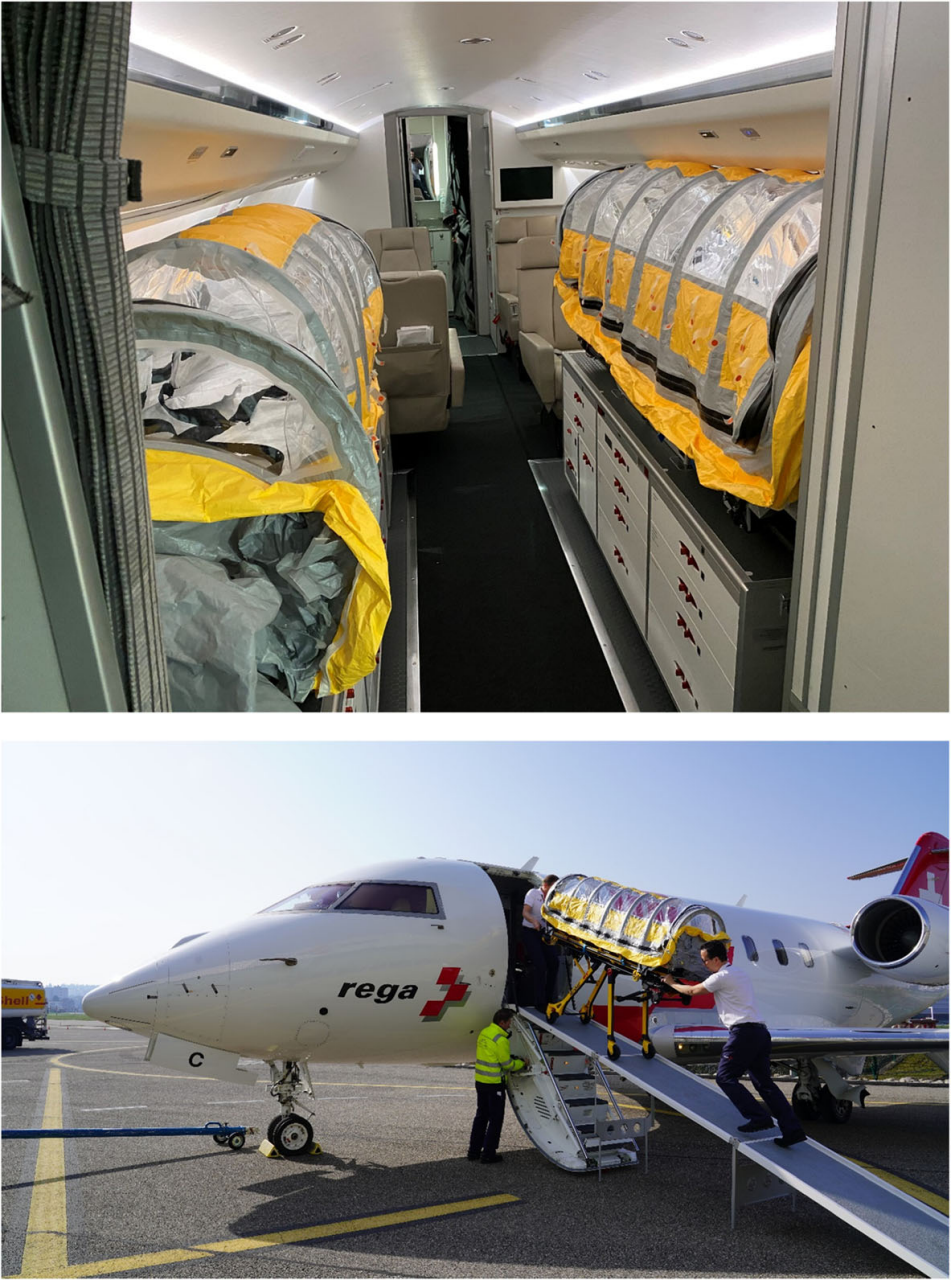
PIU owned by the REGA (©REGA - Switzerland). The REGA PIU is mainly used in the fixed wing aircrafts (Challenger 650) of the company. Transport of 2 PIU per flight are possible in the Challenger 650

Table 1 shows the hygienic measures taken to keep the risk of SARS-CoV-2 infection as low as possible for both the aeromedical crew and patients. Because there were significantly more confirmed COVID-19 patients in interfacility transport, there were also significant differences in the hygienic measures reported, except for the final disinfection of the aircraft. During the missions, none of the aeromedical crew members were infected with SARS-CoV-2. Forty-three patients were transported using a PIU, including 23 in the EpiShuttle® (Fig. 2) and 20 in the Rega-owned PIU (Fig. 3).

## Discussion

Ground emergency medical services and HEMS are part of the medical chain in the care of patients with COVID-19. Due to the pandemic, they are faced with new challenges, and different countries and health care systems vary in their reactions to the problem. Our study’s data demonstrate that, although care and transport of COVID-19 patients may be handled in different ways in different European countries, it is nevertheless safe. As the number of patients with COVID-19 could increase, however, aeromedical crews must be able to adapt.

### Preparation of COVID-19 missions

All participating air ambulance providers developed special procedures and safety instructions to be followed when transferring COVID-19 patients, including equipping the aircrafts with full PPE, and training their crews in its proper use. This and the training of crews in how to deal with COVID-19 patients could have contributed to the fact that no COVID-19 infections were reported in crew members flying these missions. During the “hot period” of the pandemic, it was difficult to get supply of PPE and disinfectant on short term basis, but the involved air ambulance providers never went out of stock.

### Mode of transport

At present, ground transport of COVID-19 patients seems to be the preferred mode of transport when caring for patients in a primary mission. This data is in line with recommendations across Europe [10]. On the contrary, for interfacility transport, air is the preferred mode. Both strategies have advantages and disadvantages. Finding the balance between ground and air transport is a challenge for the HEMS crews.

In a primary mission for a noncritical patient who needs further medical treatment in a hospital, transport by ground ambulance without physician attendance is practical, safe and resource sparing. In the case of critically ill patients, the HEMS team has to decide whether an HEMS physician should accompany the patient and which mode of transport is most useful, taking into account the distance to the hospital, possible changes of equipment, potential deterioration of the patient during transport, and the need for final disinfection. After air transport of a COVID-19 patient, the aircraft is out of service until the final disinfection, if no PIU was used.

Patients in critical condition who are being transferred to a higher-level care facility may need medical interventions en route. Air transport is the logical method in many of these cases. Data in the literature show that patients who are transferred using expedited helicopter transfer protocols have better post-transfer survival [11]. In the near future, it is possible, that the air transport of COVID-19 suspected patients in primary missions will increase, depending on the NACA score and the critical condition of the patient.

In the study involved Scandinavian countries, HEMS transport of COVID-19 patients is avoided, with the search and rescue (SAR) service of the armed forces being in charge instead. Denmark, for example, like many other European countries, has a tight network of ground-based pre-hospital critical care services, and rapid-response vehicles are staffed with consultant anaesthetists and paramedics. Most COVID-19 patients in Denmark were transported by these services in collaboration with the local EMS. Patients from islands not attached to the Danish mainland who are suspected of having or confirmed to have COVID-19 are as a rule transported in the Merlin 101 SAR helicopters. These are operated by the Danish Air Force, with critical care capabilities provided by physician-paramedic teams from the Central Denmark Region. These teams are using the EpiShuttle® to isolate patients during transport. In Norway, the procedure of dealing with COVID-19 patients is similar to Denmark.

### Cooperation among countries

In some countries, the number of serious cases was beyond what the health system could accommodate while still maintaining high European standards of care. Accordingly, some serious cases were transported to neighbouring European countries with additional intensive care capacity. Long-distance transport was primarily performed by fixed-wing aircraft, as was repatriation of European inhabitants from overseas. Especially the REGA and LAR fixed wing air ambulance services transported COVID-19 patients from south of Europe (Spain, Italy, and France) to central European countries like Germany, Switzerland, Luxembourg. The DRF-Luftrettung used EpiShuttel® equipped helicopters for cross-border transport between France and Germany. Figure S1 shows the cross-border interfacility transports of the study involved air ambulance providers.

### Medical interventions

Many medical interventions were carried out in emergency situations, such as airway management and suction which are associated with high risk of contamination. Therefore, it is of utmost importance to equip the EMS and HEMS with PPE to wear when taking care of patients with suspected or confirmed COVID-19. Modern air ambulance services are able to provide advanced interventions during transport, including intensive care procedures, differentiated catecholamine therapy, volume resuscitation, modern ventilation strategies, ECMO, inhaled nitric oxide, or even the use of heart-assisted devices (e.g. like Impella®) during transport [12–15]. Several guidelines recommend the use of ECMO or inhaled nitric oxide in selected COVID-19 patients. Some patients may deteriorate quickly and need ECMO during transport (Table 1) [16, 17].

Fluid therapy and drug administration are common in emergency medicine and intensive care, and are performed in COVID-19 patients as well. Surprisingly, the use of lung ultrasound in the pre-hospital setting is very limited, although it could help diagnose emergency patients with dyspnoea. Portable point-of-care ultrasound (POCUS) is a safe and effective bedside pre-hospital tool which can be used for initial evaluation and management in patients with confirmed or suspected COVID-19 infection [18]. The data of our survey show that even in modern, well-equipped European air ambulance systems with highly trained specialists, the potential for improvement in pre-hospital POCUS exists.

### Infection status and hygiene

Due to lack of information on infection status and limited resources and space, the risk of virus transmission in a helicopter or airplane is possibly higher than for in-hospital healthcare providers. Furthermore, many medical interventions increase the risk of virus transmission [5–8]. Examples are non-invasive ventilation (NIV), airway management, CPR, or even recording an electrocardiogram (ECG) [8, 19, 20]. Similar to SARS-CoV-1 patients, there are some COVID-19 patients who are “superspreaders” with a very high transmission rate [8]. Therefore, it is of utmost importance to equip the EMS and HEMS with PPE to wear when taking care of patients with suspected or confirmed COVID-19. Appropriate PPE is therefore recommended [21]. If information about potential COVID-19 has not been provided, initial assessment, if possible, should begin from a distance of at least 2 m from the patient [21]. During medical care, a facemask should be worn by the patient, and if transport is necessary, the number of healthcare providers in the patient compartment should be limited to a minimal number [21]. PPE should be matched to the potential mode of viral transmission, including contact (gloves, apron), droplet (gloves, apron, eye protection, FFP 2/N95 mask), or airborne (gloves, fluid-repelling long-sleeved gown, eye protection, FFP 3 mask) [21–23].

Interfacility transport missions can be very exhausting and physically stressful for the medical team. They often involve several hours of work in full PPE, conditions which may contribute to medical errors [24]. Therefore COVID-19 patients are increasingly being transferred in fixed-wing aircraft and helicopters containing PIUs like the EpiShuttle® or the Rega PIU. Due to the SARS-CoV-2 pandemic, some providers (like the DRF-Luftrettung) have equipped several HEMS bases with PIUs for safe transport of confirmed COVID-19 patients. As a result of the effective protection of the HEMS crew during transport of SARS-CoV-2 patients, helicopters do not have to be disinfected after patient transport if a PIU is used. Accordingly, the aircraft can remain in operation longer.

### Limitations of the study

Our study has some limitations with a relatively short study period in a fast changing pandemic situation*. First,* it presents a selection of European HEMS providers, and cannot be generalized for all European air ambulance services. All studied services were physician staffed and the results may not apply to non-physician staffed services. *Second*, not all invited providers were able to report COVID-19 patients’ mission-related data because COVID-19 patients were transported by the SAR service of the armed forces in their country. We do not have any information about patients’ comorbidity or hospital mortality. *Third,* European countries with a very high incidence of COVID-19, like Italy, France and Spain, did not participate in this study. Nevertheless, this study clearly shows that safe care during long transport of COVID-19 patients is achievable.

## Conclusions

Participating air ambulance providers were prepared in advance for COVID-19-related missions. Safe care and transport of suspected or confirmed COVID-19 patients is achievable. Most patients transferred after on-scene calls are transported by ground. These patients tend to be less sick than patients being transferred from one facility to another, whom needed significantly more mission-related interventions. Air transport was the preferred method for these patients, and long-distance flights were carried out by fixed-wing aircrafts.

## Supplementary information

**Additional file 1.**

**Additional file 2: Table S1.** Information regarding the air rescue service of the participating countries.

## Data Availability

The dataset generated and analysed during the current study are not publicly available due the ownership of the different air ambulance providers but are available from the corresponding author on reasonable request.

## Abbreviations

ACCD: Automated chest compression device
CE 1789: European Union standard for ambulances
COVID-19: Coronavirus disease 19
CPR: Cardiopulmonary resuscitation
EC: Eurocopter
ECG: Electrocardiogram
ECMO: Extracorporeal membrane oxygenation
EMS: Emergency medical service
FFP: Filtering face piece
H: Airbus helicopter
HEMS: Helicopter emergency medical service
i.v.: Intravenous
NACA: National Advisory Committee for Aeronautics
NIV: Non-invasive ventilation
PIU: Patient isolation unit
PPE: Personal protective equipment
SAR: Search and Rescue
SARS-CoV-2: Severe acute respiratory syndrome coronavirus 2
SD: Standard deviation
va: Veno-arterial
vv: Veno-venous
