# Supplementary material for: Pre-hospital care & interfacility transport of 385 COVID-19 emergency patients: an air ambulance perspective
Source: Scand J Trauma Resusc Emerg Med. 2020 Sep 22;28:94. doi: 10.1186/s13049-020-00789-8 (PMC7506825; doi:10.1186/s13049-020-00789-8)
Supplement: Supplementary file 1 — Additional file 1. [file 13049_2020_789_MOESM1_ESM.pptx]

## Slide 1
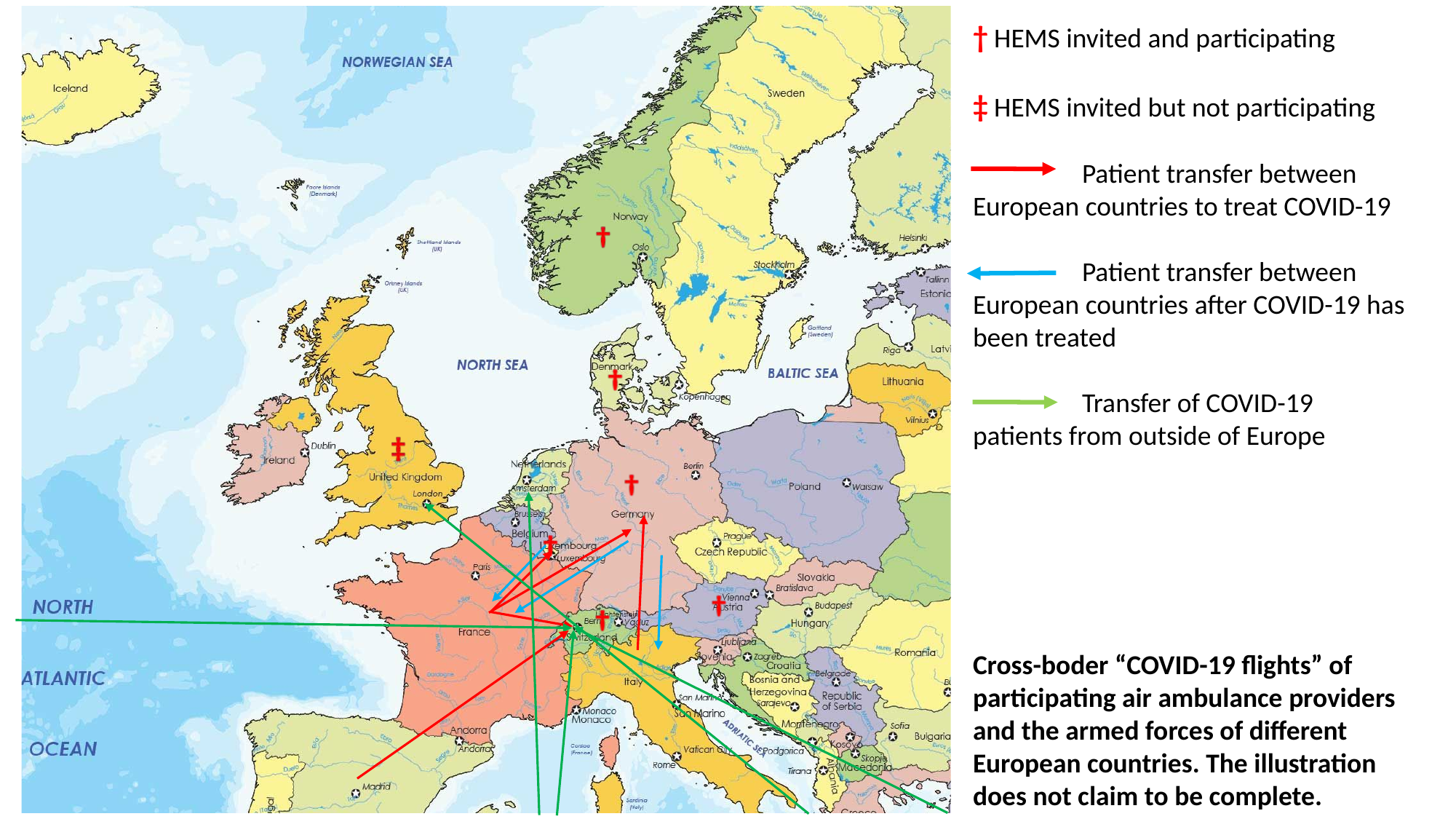

† HEMS invited and participating
‡ HEMS invited but not participating
	Patient transfer between European countries to treat COVID-19
	Patient transfer between European countries after COVID-19 has been treated
	Transfer of COVID-19 patients from outside of Europe
Cross-boder “COVID-19 flights” of participating air ambulance providers and the armed forces of different European countries. The illustration does not claim to be complete.
