# Supplementary material for: Pre-hospital care & interfacility transport of 385 COVID-19 emergency patients: an air ambulance perspective
Source: Scand J Trauma Resusc Emerg Med. 2020 Sep 22;28:94. doi: 10.1186/s13049-020-00789-8 (PMC7506825; doi:10.1186/s13049-020-00789-8)
Supplement: Supplementary file 2 — Additional file 2: Table S1. Information regarding the air rescue service of the participating countries. [file 13049_2020_789_MOESM2_ESM.docx]

**Table S1) Information regarding the air rescue service of the participating countries.**

**Austria:** The Austrian air rescue service consists of different air ambulance providers and the country has almost 9.0 million inhabitants. About 40 HEMS bases distributed over the country are operated by the different HEMS providers. The high number of HEMS bases is due to the geography of the country dominated by the Alps.

**Denmark:** The country has almost 6 million inhabitants. The Danish Public Health Authority operates 4 HEMS bases. The air rescue service of the country is supported by the SAR service of the armed forces.

**Germany:** The German air rescue service consist of different HEMS providers and the country has 83 million inhabitants. About 90 HEMS bases are distributed over the country to reach any emergency within 10-15 minutes. The air rescue service of the country is supported by the SAR service of the armed forces if necessary.

**Luxembourg:** The country has almost 630.000 inhabitants and the Luxembourg Air Rescue service is the only provider of the country. The LAR operates 3 HEMS bases and a fixed wing aircraft service.

**Norway:** The country has almost 5.5 million inhabitants. The Norwegian Air Ambulance service and the SAR service of the armed forces are in charge of the air rescue service in Norway. 12 out of 12 rotor wing aircraft bases and some fixed wing aircraft bases are operated by the Norwegian Air Ambulance.

**Switzerland:** The country has almost 8.85 million inhabitants and is covered by 17 HEMS bases. These 17 bases are operated by the different HEMS providers but mainly by the Rega.

The air rescue service in the central European countries is a cross-border service, meaning that rescue helicopters from neighboring countries fly across the border to emergencies if needed.
